# A Wi-Fi-Based Passive Indoor Positioning System via Entropy-Enhanced Deployment of Wi-Fi Sniffers

**DOI:** 10.3390/s23031376

**Published:** 2023-01-26

**Authors:** Poh Yuen Chan, Ju-Chin Chao, Ruey-Beei Wu

**Affiliations:** Department of Electrical Engineering and Graduate Institute of Communication Engineering, National Taiwan University, Taipei 10617, Taiwan

**Keywords:** Internet of Things, Wi-Fi-based passive indoor positioning system, Wi-Fi Sniffer, received signal strength indicator

## Abstract

This study presents a Wi-Fi-based passive indoor positioning system (IPS) that does not require active collaboration from the user or additional interfaces on the device-under-test (DUT). To maximise the accuracy of the IPS, the optimal deployment of Wi-Fi Sniffers in the area of interest is crucial. A modified Genetic Algorithm (GA) with an entropy-enhanced objective function is proposed to optimize the deployment. These Wi-Fi Sniffers are used to scan and collect the DUT’s Wi-Fi received signal strength indicators (RSSIs) as Wi-Fi fingerprints, which are then mapped to reference points (RPs) in the physical world. The positioning algorithm utilises a weighted k-nearest neighbourhood (WKNN) method. Automated data collection of RSSI on each RP is achieved using a surveying robot for the Wi-Fi 2.4 GHz and 5 GHz bands. The preliminary results show that using only 20 Wi-Fi Sniffers as features for model training, the offline positioning accuracy is 2.2 m in terms of root mean squared error (RMSE). A proof-of-concept real-time online passive IPS is implemented to show that it is possible to detect the online presence of DUTs and obtain their RSSIs as online fingerprints to estimate their position.

## 1. Introduction

Wi-Fi is a crucial element of daily life and is present in most indoor environments. RF signals for IoT applications can be used to produce an inexpensive and accurate low-cost indoor positioning system [1]. This study creates a positioning system with acceptable accuracy without the need for additional hardware deployment, so it is cost-effective [2,3,4]. Indoor positioning systems (IPS) have gained increased attention in recent years as people spend more time indoors, and the Covid-19 pandemic has prompted or required people to stay indoors more frequently. Indoor location-based services (ILBS) have the potential to become an integral part of daily life, not only for traditional indoor wayfinding, proximity advertisements, and accurate contact tracing, but also for applications such as building emergency management [5], smart energy management [6], smart HVAC controls [7], elderly monitoring, and crime prevention.

Indoor positioning systems (IPS) present a challenge due to the complexity and dynamic nature of indoor environments, in contrast to GPS, which is used for outdoor positioning. Other radio signals, such as Bluetooth Low Energy (BLE) [8,9], Radio Frequency Identification (RFID) [10], and Ultra-wideband (UWB) [11], have also been used for IPS. However, BLE has a slower transmission speed than Wi-Fi and is less suitable for real-time tracking of moving devices. Passive RFID tags are cost-effective but have a limited transmission range, requiring more tags to be installed and maintained than a single Wi-Fi access point (AP). Active RFID tags have a more extended range but are more expensive to implement. UWB has the potential for higher positioning accuracy with large bandwidth and fast data transmission but requires additional hardware deployment.

In this research, we focused on developing a Wi-Fi-based IPS. Wi-Fi is the most commonly used technology for indoor network connections and is widely integrated into modern wireless devices such as smartphones. The additional deployment of hardware such as BLE, RFID, and UWB for indoor positioning alone may not justify the cost of implementation and maintenance. It may require users to carry a separate signal transmitter/receiver. This defeats the purpose of passive positioning. Therefore, Wi-Fi-based IPS is a practical and cost-effective solution for real-time development.

The information in Wi-Fi signals includes the received signal strength indicator (RSSI). This measures the attenuation of an RF signal as it propagates from the transmitter to the receiver. In real-world environments, there is no single direct path. Radio wave propagation is subject to reflection, scattering, and diffraction due to obstructions, which are known as multipath effects. Therefore, the RSSI depends on environmental conditions. A RSSI fingerprinting technique is used to associate signal information with physical space. Previous studies developed heterogeneous systems using multi-source fusion, such as pedestrian dead reckoning (PDR), with RSSI fingerprints [12], and video with RF signals for real-time positioning [13]. The device-under-test (DUT), particularly the smartphone, actively participates in the positioning system through the installation of additional application programming interfaces (APIs). Therefore, complementary features are used to increase localisation positioning accuracy. This is known as an active Wi-Fi-based indoor positioning system (IPS).

For passive Wi-Fi-based IPS, surrounding Wi-Fi Sniffers scan and collect information from the DUT to calculate the position. For example, the DUT (smartphone) is connected to nearby Wi-Fi for Internet access, but not accessing any application for real-time positioning. In this case, since the DUT is not actively participating in the positioning process, only a limited amount of information can be obtained to identify and track the DUT. Additional information such as PDR is not available due to operating system restrictions. Wi-Fi Sniffers in the surrounding can obscurely detect the presence of nearby Wi-Fi devices such as the DUT by capturing Wi-Fi packets. This is known as network-side fingerprinting.

Previous work on passive fingerprinting can be categorised into using Wi-Fi probes and Wi-Fi data frames, with the latter having the advantage of collecting RSSI in high density under the same amount of time. Other than the type of Wi-Fi information, the placement of Wi-Fi Sniffers should be investigated so that they can be deployed optimally to increase positioning accuracy. For passive Wi-Fi IPS, increasing the positioning accuracy of the DUT remains a challenge, along with its identification. While works in [14,15] have successfully realised a passive fingerprinting system with the help of an active fingerprinting radio map to reduce missing RSSI values, to our best knowledge, this is the first study to incorporate the optimal placement of Wi-Fi Sniffers into consideration for Wi-Fi-based passive fingerprinting implementation in the real world in order to reduce the positioning errors, which can be a baseline for future related study.

The major achievements of this study include the following:

1. It proposes the use of a Genetic Algorithm (GA) with an entropy-enhanced objective function to determine the optimal deployment of Wi-Fi Sniffers in the area of interest for indoor positioning.
2. Real Wi-Fi Sniffers are deployed to implement passive IPS, which identifies a DUT by finding the DUT’s MAC address from a pre-deployed Wi-Fi access point (AP).
3. The real implementation of a real-time online passive indoor positioning system (IPS) as a proof of concept. The IPS detects the online presence of DUTs and uses their RSSIs as online fingerprints for position estimation.

The remainder of the study is organised as follows. Section 2 presents related work for passive IPS. Section 3 describes the simulation of the Wi-Fi RSSI. Section 4 optimises the deployment of Wi-Fi Sniffers. Section 5 describes the automated collection of Wi-Fi fingerprints. Section 6 determines the positioning errors for simulated and measured data. Section 7 discusses the online IPS, and Section 8 draws conclusions and gives details of future work.

## 2. Related Work

Active Wi-Fi-based IPS has certain disadvantages. Installing additional APIs on the DUT, such as in [16], may not be practical, particularly for military and commercial applications. Active Wi-Fi scanning also consumes resources from the DUT and drains the battery more quickly, which may not be desirable for the user. [17].

Wi-Fi signals are theoretically bi-directional and reciprocal, which means that a Wi-Fi module can run in monitor mode to collect information from the DUT and run in access point mode to provide internet service. However, currently, no commercially available Wi-Fi APs support operating in access point mode and monitor mode simultaneously, so a custom Wi-Fi Sniffer device is required to collect information such as RSSI, Channel State Information (CSI), or Probe request message (PRqM) from the DUT. Additionally, suppose the DUT is not actively transmitting its Wi-Fi information to nearby Wi-Fi APs. In that case, detecting and tracking the DUT in real-time can be challenging using network-side fingerprinting techniques for indoor positioning.

**Figure 1:**
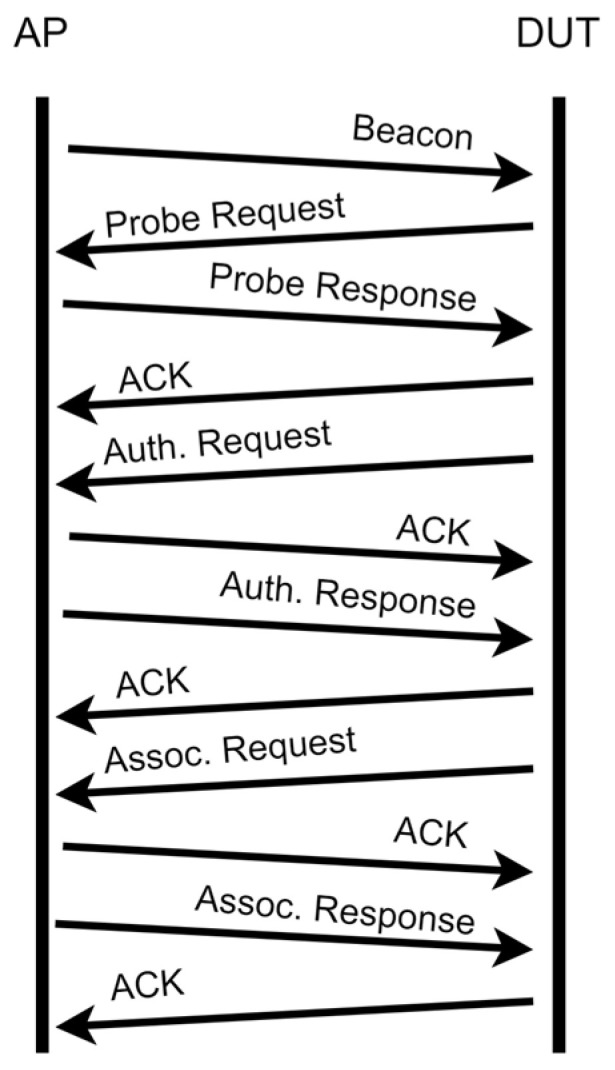
The 802.11 association process [18].

Figure 1 shows the 802.11 association process [18]. The PRqM is used to passively detect the presence of APs in the surroundings [19,20,21]. A DUT sends PRqMs periodically over multiple Wi-Fi channels, approximately every 10–15 seconds, to discover nearby Wi-Fi APs. The PRqM contains the MAC address of the DUT for identification and RSSI as Wi-Fi information. When the connection is established, the DUT can specify a particular SSID to ensure a seamless connection. This message is sent occasionally from a DUT even if Wi-Fi is disabled.

One study [20] implemented an active request-to-send (RTS) packet injection to force the DUT to reply with a clear-to-send (CTS) message and increase the updating rate. This increases the number of Wi-Fi packets from the DUT to allow Sniffers to capture them faster.

Some studies use RSSI as Wi-Fi fingerprints for passive positioning. One study [22] used a passive Wi-Fi tracking system to track DUTs in an exhibition venue. An Expectation-maximisation (EM) algorithm determines the optimal parameters for the signal-strength-to-distance model for different DUT models to reduce the positioning error. The system determines the MAC address of the DUT via a Wi-Fi connection and 10 Wi-Fi Sniffers were deployed in the experimental venue. The study showed that different DUT models produce different positioning errors, of as much as 5.3 m if fingerprinting is used and as low as 1.7 m if multi-lateration is used. The study did not determine the optimal deployment for Wi-Fi Sniffers.

In terms of passive systems, Wi-Fi Sniffers can miss Wi-Fi packets from the DUT because the DUT does not actively participate in the process. Wi-Fi Sniffers may also not cover specific reference points (RPs) (dead zone) because the signal from the DUT is weak. Building structures that are not suitable for the installation of Wi-Fi Sniffers also degrade positioning performance. To overcome the above-mentioned shortage, Ko et al. [15] used legacy active Wi-Fi fingerprints as complementary features to enhance passive fingerprints. Eight Sniffers were used for passive fingerprinting in the experimental venue; using KNN, the average positioning error is 2.18 m. In addition, Luo et al. [23] proposed passive IPS using the mapping method for the collected RSS traces to specific indoor pathways by placing the Sniffers in evenly.

The placement of access points or sniffers is a critical factor impacting user transmission experience and localisation services in passive Wi-Fi-based IPS systems. Previous studies of passive Wi-Fi-based IPS deal with the extraction of Wi-Fi information from unmodified DUTs for positioning, increasing the number of Wi-Fi packets by injecting packets, and reducing positioning error using various positioning algorithms. Elmosilhy et al. [24] use GA to maximise the total system capacity and minimise transaction error. However, little attention has been paid to the effect of different deployment patterns for Wi-Fi Sniffers on positioning accuracy.

Ismail et al. [25] proposed an optimised deployment method considering the percentage of dead zones, the average strength of RSSI, and the variance of the power of RSSI in each RP. Ibrahim et al. [26] compared different objective functions in the genetic algorithm and the quantity of the access points on the localisation error. However, according to our experiment, the more Sniffers we deployed, the more the estimated deployment lead Sniffers to be thronged. The standard GA sometimes produced solutions where multiple Sniffers were placed close to each other. In other words, the features of the RP in several Sniffers would have the same trends and cause the part to be redundant. This can be problematic because the Sniffers should be distributed evenly to provide the maximum amount of distinct and useful information. We proposed the Entropy-Enhanced objective function to address this problem, elaborated in Section 4.

## 3. Simulation: Wi-Fi RSSI Simulation Using iBwave Wi-Fi Suite

In homes and small and medium enterprises and other unmonitored indoor premises, Wi-Fi APs are randomly placed to ensure good wireless communication. Wi-Fi signals are strong or weak at various RPs, so for indoor positioning, it is troublesome. Passive Wi-Fi indoor positioning systems do not have access to additional complementary features for real-time positioning without the active endorsement of the DUT. It is essential to extract the maximum amount of useful information from each Sniffer on each RP to ensure maximum positioning accuracy.

Some modern institutions and venues, such as hotels and shopping malls, use a site surveying tool that contains information about the floor plan to deploy Wi-Fi infrastructure. For example, iBwave [27], which is a provider of commercial network design solutions, lists some case studies of the optimal design and deployment of Wi-Fi networks in airports, campuses, and convention centres, on its official website. The company designs and visualises signal propagation for Wi-Fi networks in 3D by predicting signal coverage and wireless performance for indoor wireless networks.

**Figure 2:**
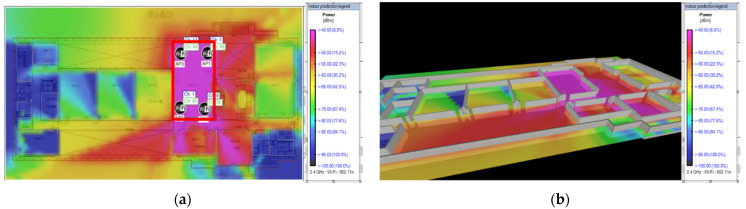
RSSI simulated by iBwave. (**a**) Simulated Wi-Fi 2.4 GHz RSSI for all APs in an area of interest using iBwave; (**b**) 3D modelling for simulated Wi-Fi 2.4 GHz RSSI of all APs in an area of interest using iBwave.

The fast ray tracing propagation model is a default choice. The simulation tool allows floor plans that are scaled to real-world measurements, and walls that are constructed of different materials and are of different heights are modelled to better represent the real-world environment. Figure 2a shows the overall predicted Wi-Fi signal strengths for AP0, AP1, AP2, and AP3 in the Wi-Fi 2.4 GHz band at a height of 3 m. In the heatmap, pink indicates a stronger RSSI and dark blue indicates a weaker RSSI. Figure 2b shows a 3D counterpart with a better view of modelling the wall.

**Figure 3:**
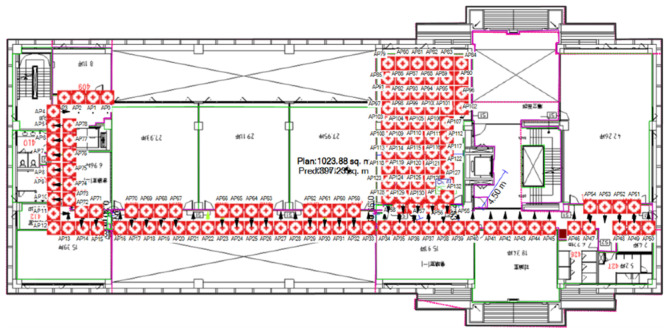
Wi-Fi Sniffers are deployed at R possible locations in the area of interest in the using simulation tool.

Figure 3 shows the Wi-Fi infrastructure, as deployed using the simulation tool, which identifies R possible locations for Sniffers before deployment. In this setup, it is assumed that the simulated signal strength for the Wi-Fi AP is equivalent to the RSSI that is measured by the Wi-Fi Sniffer that is deployed in the real world, so the Wi-Fi AP for this simulation context is treated as a Wi-Fi Sniffer. We investigate the correlation between the simulated signal strengths by the Wi-Fi AP and the RSSIs monitored by the Wi-Fi Sniffer deployed in the real world in the following experiments. Furthermore, the estimated signal strengths of all Sniffers on each RP by the iBwave software would form a pseudo fingerprint feature for the Sniffer deployment.

## 4. Methodology: Optimal Deployment of Wi-Fi Sniffers Using Genetic Algorithm and Entropy-Enhanced Genetic Algorithm

Each Wi-Fi on different RPs must provide distinct features so that the information is helpful to distinguish different RPs for positioning algorithms. If M Sniffers are deployed in the experimental venue, M Sniffers must be optimally selected from the R possible Wi-Fi Sniffers locations so that at each RP, the RSSI for at least $K_{S}$ Sniffers can be used for positioning.

**Figure 4:**
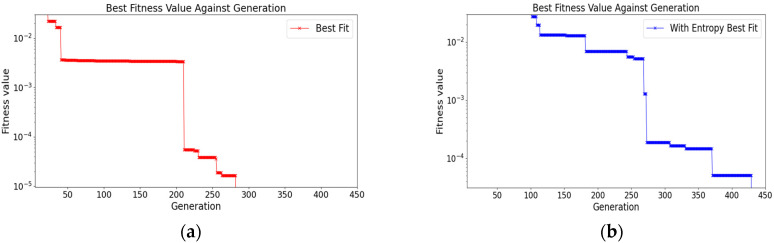
Figures of convergences of the solutions. (**a**) Convergence of the solution using the GA; (**b**) Convergence of the solution for a GA using entropy.

The placement of Wi-Fi Sniffers is a constrained optimisation problem. The classic Genetic Algorithm (GA) is adopted to explore the state-space landscape to look for the optimal point instead of checking through all the deployment permutations. This study uses a set of R=133 possible Wi-Fi Sniffers locations $\left(L_{1},L_{2},\ldots ,\;L_{R}\right)$, as shown in Figure 4. M=20 Wi-Fi Sniffers are deployed in these R locations, $K_{S}$ has a value of 5, and configuration Q is a set of specified locations for the Wi-Fi Sniffers. The simulated RSSIs using iBwave at RP n (*n* = 1, 2, …, *N*) due to all Wi-Fi Sniffers being combined into vector $r_{mn}=\left(RSSI_{1},\;RSSI_{2},\ldots ,RSSI_{M}\right)$ to form a fingerprint.

Similar to previous studies [25,26], a Wi-Fi Sniffer configuration selection algorithm is developed using a GA with the mean $\bar{r}$, variance $\sigma_{r}^{2}$, and penalty parameter P as variables to minimise the objective function J and obtain the optimal configuration Q for deployment through generations.

The simulation uses 524 (N) of RP. The minimum RSSI setting for the algorithm is $RSSI_{min}=$ −80 dB, because communication is not possible at lower values. The maximum RSSI is $RSSI_{max}=$ −30 dB. The raw RSSI value $r_{mn_{raw}}$ at each RP is normalised as

(1) $$r_{mn}=\frac{r_{mn_{raw}}-RSSI_{min}}{RSSI_{max}-RSSI_{min}}$$

The mean and variance for each configuration are calculated as follows:

(2) $$\bar{r}=\frac{1}{N_{T}}\overset{M}{\underset{m=1}{\sum }}\overset{N}{\underset{n=1}{\sum }}r_{mn}$$

(3) $$\sigma_{r}^{2}=\frac{1}{N_{T}-1}\overset{M}{\underset{m=1}{\sum }}\overset{N}{\underset{n=1}{\sum }}{\left(r_{mn}-\bar{r}\right)}^{2}$$

where $N_{T}=M\times N$.

The objective function J defined in [24] is

(4) $$\arg\min J=\omega_{1}\frac{1}{\bar{r}}+\omega_{2}\sigma_{r}^{2}+P$$

The weights in (4) are $\omega_{1}=0.8$ and $\omega_{2}=0.2$, similar to the values for a previous study [25]. The average RSSI at the RP is maximised and the variance is minimised. *P* is defined as the percentage of RPs in the dead zone where not all the largest $K_{S}$ RSSI values for the Sniffers are greater than $RSSI_{min}$.

The parameters for the GA are shown in Table 1. The GA produces a convergent solution. The configurations in the population are initialised. Each configuration (chromosome) consists of *M* randomly selected Wi-Fi Sniffers (genes) from *R* possible locations. The fitness value *J* for each configuration is calculated based on the objective function (4). Half of the configurations with smaller *J* values are retained in the population for the next generation. Two of these better configurations are then randomly selected and their genes are mixed to generate a new array of 2*M* locations, in which *M* locations are randomly selected to form a new configuration. This crossover process is repeated to fill the other half of the configurations that are required for the new population for the next generation.

After the crossover, the algorithm iterates over each index in the configuration to monitor mutation. If a mutation occurs, a number in the range of 1 to R (inclusive) is randomly selected to represent a new Sniffer location index in the original configuration. Using the smallest fitness value that is obtained at generation 290, the solution converges as shown in Figure 4a.

**Figure 5:**
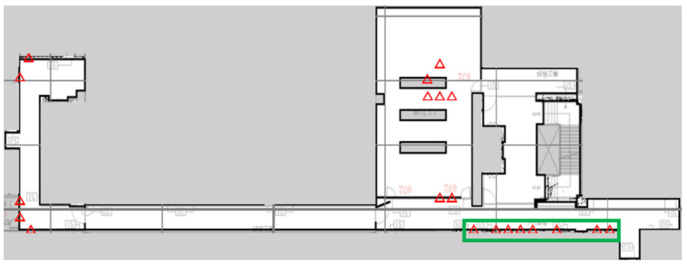
The optimal configuration Q that is calculated using the GA (20 Wi-Fi Sniffers).

The optimal deployment pattern of Wi-Fi Sniffers, as determined using GA, is shown by the red triangles in Figure 5. The GA gives a converged solution, but eight Wi-Fi Sniffers are placed in close proximity, as shown in the green box. In general, Wi-Fi Sniffers should be distant from each other so that each gives significantly distinct and useful information. Therefore, to maximise the contributions of each Wi-Fi Sniffer to the maximum number of RPs, entropy S is used to enhance the objective function of the GA in this study. Entropy is a common metric for machine learning because it measures the unpredictability or impurity of a system [28,29,30,31,32]. The more disordered or impure a feature is, the greater the amount of information that can be extracted from that feature to better discriminate between Sniffers.

Before defining the entropy S for GA, it is important to use the concept of the covariance matrix for principal component analysis (PCA) to determine the correlation between Wi-Fi Sniffers [33,34]. An M×M covariance matrix Σ**,** for which each element represents the covariance between Sniffers, is defined as follows:

(5) $$\Sigma_{ij}=\sigma \left(r_{i},r_{j}\right)=\frac{1}{N-1}\overset{N}{\underset{n=1}{\sum }}\left(r_{in}-\bar{r_{i}}\right)\left(r_{jn}-\bar{r_{j}}\right)$$

In matrix form, this is written as follows:

(6) $$\Sigma ={\left[\begin{array}{cccc} \Sigma_{11} & \Sigma_{12} & \ldots & \Sigma_{1M}\\ \Sigma_{21} & \Sigma_{22} & \ldots & \Sigma_{2M}\\ \vdots & \vdots & \ddots & \vdots \\ \Sigma_{M1} & \Sigma_{M3} & \cdots & \Sigma_{MM} \end{array}\right]}_{M\times M}$$

where $\Sigma_{11}$ represents the self-variance of Sniffer 1 and $\Sigma_{12}$ represents the covariance between Sniffers 1 and 2.

The eigenvalues λ of Σ are calculated to determine the variance in the data along the new feature axes, only if vector A, which is the eigenvector of Σ that corresponds to the eigenvalue λ, is non-zero, so:

(7) ΣA=λA

where the eigenvalues $\lambda =\lambda_{1},\lambda_{2},\ldots ,\lambda_{M}$ are sorted in descending order. The sum of these eigenvalues is $\lambda_{\mathrm{total}}$ The normalised eigenvalues are defined as ${\tilde{\lambda }}_{m}=\lambda_{m}/\lambda_{\mathrm{total}}$ for m=1,⋯, M such that each has a value between 1 and 0. Entropy is then defined as follows:

(8) $$S=-\overset{M}{\underset{m=1}{\sum }}{\tilde{\lambda }}_{m}\;\cdot \;log_{2}{\tilde{\lambda }}_{m}$$

which are used to measure the correlation between the Sniffers. By maximising the entropy, the GA encourages a more even distribution of the Sniffers and helps avoid having multiple Sniffers placed in close proximity to each other.

For the optimal condition, each value for λ is equal. There is no correlation between the Sniffers, so their contributions to the features are equally important, and the covariance matrix is a unit matrix. However, in a real environment, the positions of Sniffers affect the covariance matrix. If Sniffers are too close to each other, their features are more correlated so some of the information that they provide is less useful.

The entropy-enhanced objective function is defined as follows:

(9) $$\arg\min J=\omega_{1}\frac{1}{\bar{r}}+\omega_{2}\overset{2}{\underset{r}{\sigma }}-\omega_{3}S+e^{\omega_{4}P}$$

where subtraction is performed on entropy value to maximise its contribution. An exponent is also used for the penalty parameter P to increase its sensitivity in terms of penalising the dead zone. The parameter values in Table 1 are used to calculate the converged solution using the entropy-enhanced objective function in the GA. The solution is obtained at generation 430, as shown in Figure 4b.

**Figure 6:**
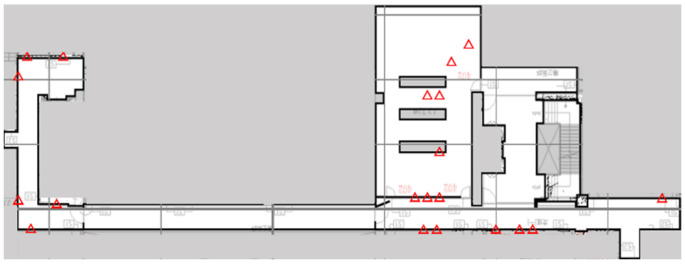
The optimal configuration Q using the GA with entropy (20 Wi-Fi Sniffers).

Figure 6 shows the optimal positions of Sniffers using the entropy-enhanced objective function. The Sniffers are further from each other than those in Figure 5, because S is included in the calculation. The optimisation is repeated several times using different initial conditions, and the results show that the positions of Sniffers do not differ significantly. The configuration with the smallest fitness value for these trials is used for the real deployment.

## 5. Experimental Setup: Automated Collection of Wi-Fi Fingerprints as Offline Data

**Figure 7:**
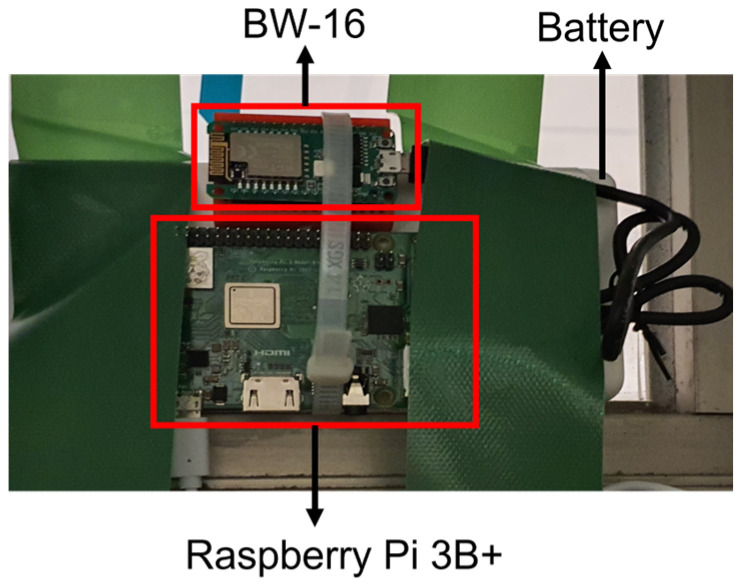
Device configuration for the real-world experiment.

Multiple Realtek BW-16s are deployed as Wi-Fi Sniffers to scan and collect information from nearby Wi-Fi-enabled devices. The BW-16 is embedded with one Wi-Fi radio module and can operate in Wi-Fi 2.4 GHz and 5 GHz bands. When working in Wi-Fi monitor mode, it is impossible to operate in Wi-Fi client mode to connect to the network for data transfer between the endpoint and server. Therefore, a suitable Wi-Fi gateway is needed to transfer and receive data. In the study, Raspberry Pi 3B+ is chosen as the Wi-Fi client for BW-16. This fully functional single-board computer is incorporated with a Wi-Fi module and completed with decent processing power and memory for remote programming and control, as well as supporting general-purpose input/output (GPIO) for system expansion. As real-world deployment often involves proper power supply with wiring for the Wi-Fi infrastructure, the Wi-Fi Sniffer is connected to a portable power supply, as shown in Figure 7 in this preliminary study.

**Figure 8:**
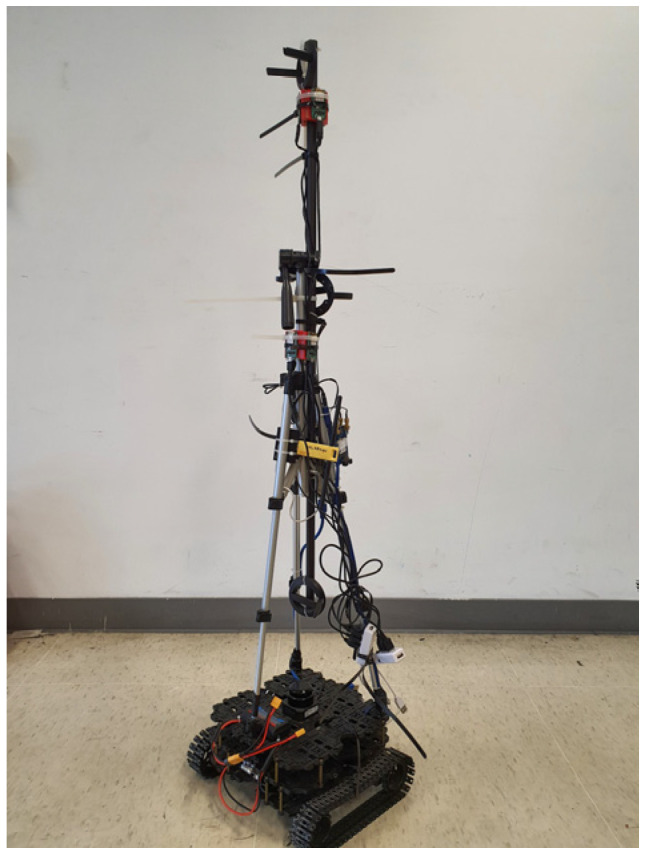
The in-house surveying robot that automatically surveys the Wi-Fi site.

Using an in-house Wi-Fi surveying robot, which is shown in Figure 8, the fingerprints for the configurations of Wi-Fi Sniffers for both Wi-Fi bands that are calculated using the entropy-enhanced objective function of GA and those that do not use entropy-enhanced objective function are collected on each RP to whether the proposed method increases positioning accuracy.

The robot is based on a TurtleBot3 with a robot operating system (ROS) 1. It uses a light detection and ranging (LiDAR) sensor, an open-source control module for the ROS (OpenCR), a mini personal computer (PC), a Wi-Fi adapter, multiple Realtek BW-16s as the DUTs, and portable batteries. The BW-16s were placed at different heights and orientations on the robot and were configured to operate as Wi-Fi DUTs, such as smartphones, for which Wi-Fi Sniffers can passively collect their Wi-Fi packets by monitoring Wi-Fi traffic in the vicinity.

**Figure 9:**
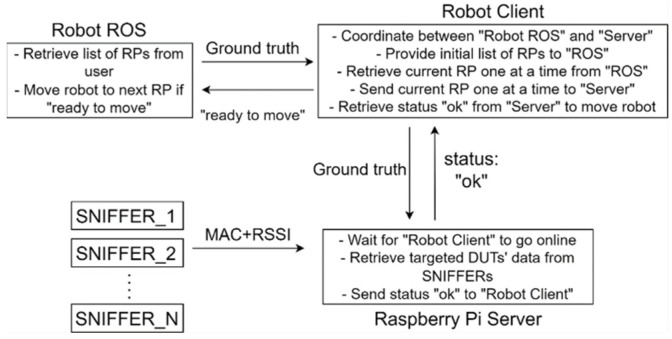
Logical flow for coordination between the robot and the server for automated collection of training data.

The logical flow of coordination between the robot and the server for the automated collection of training data is shown in Figure 9. A Raspberry Pi 4 server verifies that all Wi-Fi Sniffers are online and that the connection is sufficient for data transfer while it waits for the robot client to come online. To collect RP data for an indoor positioning system, a list of targeted RPs is input into a ROS as ground truth for the robot’s movement. A robot client program that is written in Python acts as an intermediary between the robot and the server. It transmits the current RP to the server and the server embeds the ground truth in the collected RSSI fingerprints. The server confirms that all fingerprints on the current RP have been collected by sending an “ok” signal to the robot client and the client then sends a “ready to move” signal back to ROS, which moves to the next RP.

This study uses weighted K-nearest neighbours (WKNN) for the positioning algorithm. The Euclidean distance between the fingerprint of the DUT and the fingerprints of all RPs is calculated using the normalised value for the RSSI. Position estimate $\hat{p}$ is calculated as follows:

(10) $$\hat{p}=\frac{1}{\sum_{k=1}^{K}w_{k}}\times \overset{K}{\underset{k=1}{\sum }}w_{k}p_{k}$$

where the K smallest Euclidean distances $D_{k}$ are used, $p_{k}$ are the coordinates of the k^th^ RP, and the weight $w_{k}=1/D_{k}$. The value of K is 10 [32], a trade-off for accuracy and performance which will be explained in the next Section.

**Figure 10:**
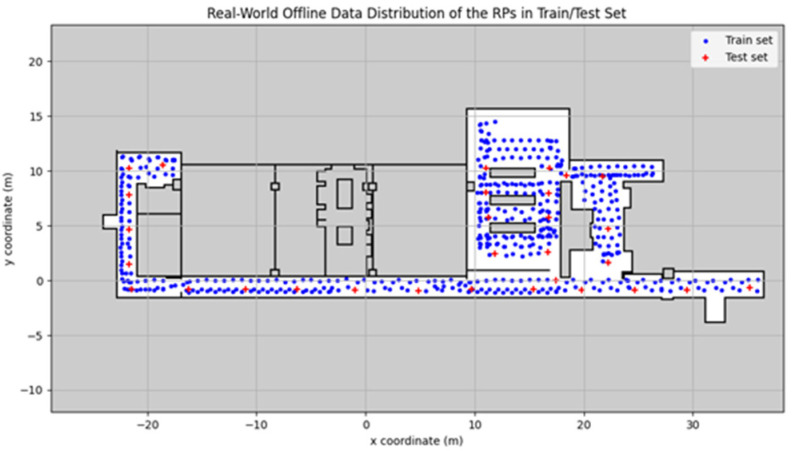
Distribution of the RPs for a train/test in a real environment for offline data with entropy.

The evaluation metric is the root mean squared error (RMSE), which is defined as follows:

(11) $$RMSE=\sqrt{\frac{\sum_{i}^{N_{0}}{\left(\hat{p_{i}}-\tilde{p_{i}}\right)}^{2}}{N_{0}}}$$

where $N_{0}$ is the total number of testing RPs and $\tilde{p}$ denotes their ground truth. Figure 10 shows the training and testing RPs using offline data with entropy, which are respectively represented by blue circles and red crosses. There are 524 training points and $N_{0}=$ 30 testing points.

## 6. Results and Discussion: Positioning Accuracy Using Simulated and Offline Measured Data

The results in Table 2 show that the RMSE for the Wi-Fi Sniffers configuration that is calculated using entropy for the Wi-Fi 2.4 GHz and 5 GHz bands is less than the value that is calculated not using entropy. For the GA optimisation, iBwave is used to calculate and simulate electromagnetic waves. The software could simulate both the 2.4 GHz and 5 GHz Wi-Fi signals. For the measurement in the Wi-Fi 5 GHz band, the RMSE value is 24.7% less, and the RMSE value for the Wi-Fi 2.4 GHz band is 7.1% less. Using the simulation data for the 5 GHz and 2.4 GHz bands, the RMSE values are 14.6% and 19.2% less, respectively, if entropy is used for the calculation.

The results show that positioning accuracy is increased using an optimised configuration with entropy enhancement, but the increase is not significant in some cases. Therefore, comparisons are made using different numbers of Wi-Fi Sniffers to determine whether this observation is valid.

**Figure 11:**
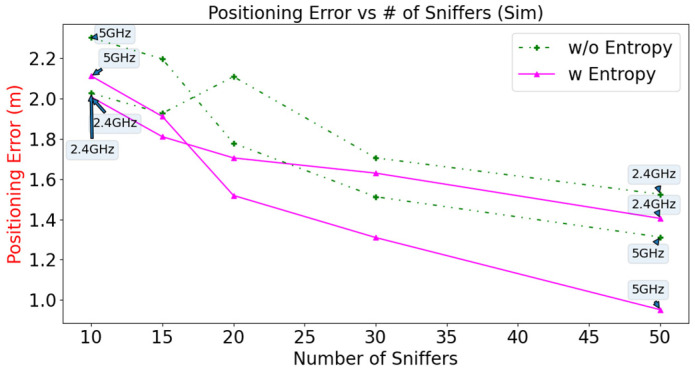
Positioning error versus number of Sniffers for different configurations of simulated data (10, 15, 20, 30, 50 Sniffers).

The positioning error using the simulated data versus the number of Sniffers is plotted in Figure 11. For 20 Wi-Fi Sniffers, configurations using entropy (2.4 GHz and 5 GHz) have a lower RMSE than configurations that do not use entropy. The corresponding RMSEs are (1.705 m and 1.519 m) and (2.109 m and 1.778 m), respectively. The configuration that is calculated using entropy for the Wi-Fi 5 GHz band has the lowest RMSE value of 0.953 m if 50 Sniffers are deployed. The use of extra 30 Sniffers increases the accuracy by 37.2%. In general, the positioning error decreases as the number of deployed Sniffers increases.

**Figure 12:**
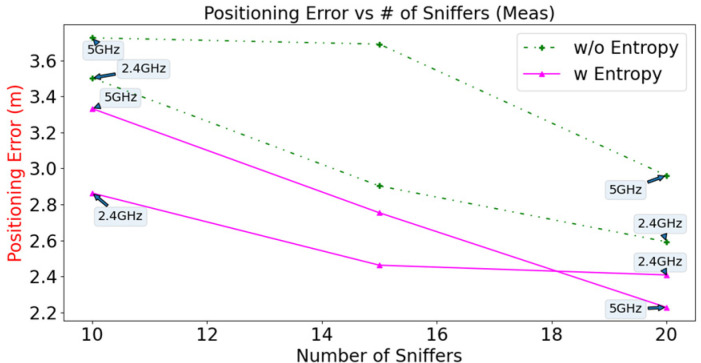
Positioning error versus number of Sniffers for different configurations of measurement data (10, 15, 20 Sniffers).

The positioning error for the measured data versus the number of Sniffers is plotted in Figure 12. For different numbers of Sniffers (10, 15, 20 Sniffers), the positioning errors for the configurations that are calculated using entropy for both Wi-Fi bands are less than those for configurations that are not calculated using entropy. The results for simulation and measurement verify that using a GA with entropy allows more accurate positioning accuracy than calculations that do not use entropy.

**Figure 13:**
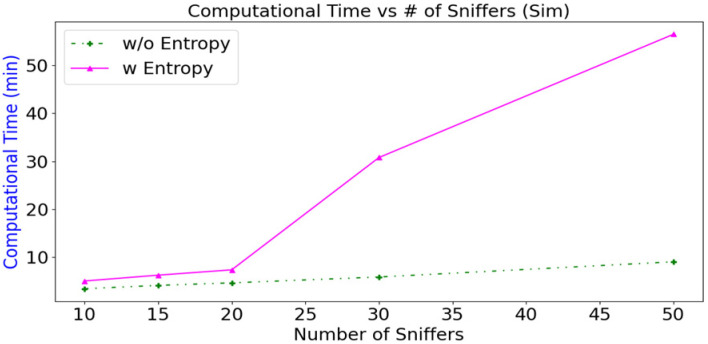
Calculation time versus number of Sniffers for different configurations of simulated data (10, 15, 20, 30, 50 Sniffers).

Figure 13 shows that generating the optimal configuration using entropy takes longer than not using entropy because time is required to calculate the covariance matrix and for eigen analysis. For configurations that use 20 Sniffers or less, the calculation time is nearly the same, whether entropy is used. However, optimising 50 Sniffers require 670% more time if entropy is used. The time that is required to calculate the optimal solution only affects the pre-deployment phase. Even though there are many permutations, the computational time for 20 Wi-Fi Sniffers is still under 10 minutes, which is acceptable.

**Figure 14:**
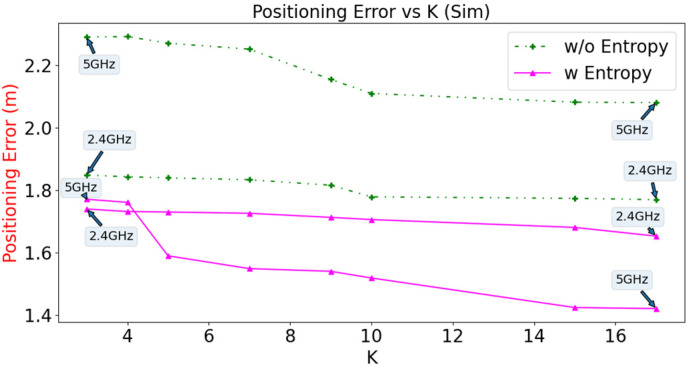
Positioning error versus the value of K for simulated data.

**Figure 15:**
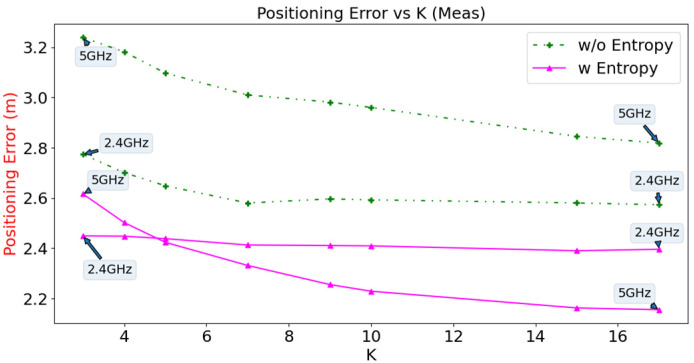
Positioning error versus the value of K for measured data.

The effects of different values of K on positioning error for the WKNN are analysed using simulated and offline measured data, with the number of Wi-Fi Sniffers fixed at 20. The results are shown in Figure 14 and Figure 15, respectively. From the simulation results, the lowest positioning error of 1.42 m is achieved using entropy in the Wi-Fi 5 GHz configuration at K = 17. For the measured data, the lowest positioning error is 2.16 m using the same configuration. In general, the configuration that uses entropy has a lower positioning error than the configuration that does not use entropy for both Wi-Fi bands for simulated and measured data. As the value of K increases, the positioning errors generally decrease until they reach a point of saturation. The optimal value of K can be determined through cross-validation. It is worth noting that higher values of K can lead to longer computation times and slower update rates in online systems. In this study, a value of K = 10 was chosen as a trade-off between positioning error and computation time.

## 7. Use Case: Online Passive Indoor Positioning

This study calculates the optimal deployment of Wi-Fi Sniffers and uses a passive Wi-Fi-based IPS in a real environment to demonstrate proof-of-concept. The fourth floor of the Ming-Da Building in National Taiwan University is the area of interest for this study. This preliminary study involves a scenario whereby a “free” Wi-Fi connection is provided, so all users can connect their DUT to that offered Wi-Fi. At the same time, the current passive IPS can extract the DUT’s MAC address for identification and its RSSI from the Sniffer for positioning. This ensures that Wi-Fi packets from the DUT are available at regular intervals, allowing the Wi-Fi Sniffers to capture the necessary information for passive positioning in real-time. Note that DUTs do not connect directly to Wi-Fi Sniffers.

**Figure 16:**
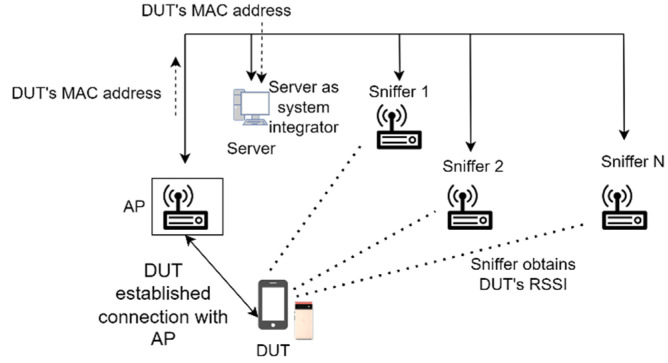
Extraction of DUT’s MAC address in this study.

Figure 16 illustrates the extraction of the DUT’s MAC address for this study. The MAC address of the DUT was extracted as follows:

1. The DUT connects with a randomly placed wireless AP.
2. The server obtains the DUT’s MAC address from the offered AP.
3. The MAC address is then sent to the wireless Sniffers to record the DUT’s RSSI.
4. The RSSI values are then sent back to the server for data integration.

By keeping a list of connected clients available on the AP, the system can quickly identify which device is being used for positioning. Even if MAC randomisation occurs on the DUT, the IPS can still detect and localise the DUT because a new MAC address will be registered to the Wi-Fi AP. However, identifying if the same DUT has been registered with a different MAC address in the past remains a challenge.

**Figure 17:**
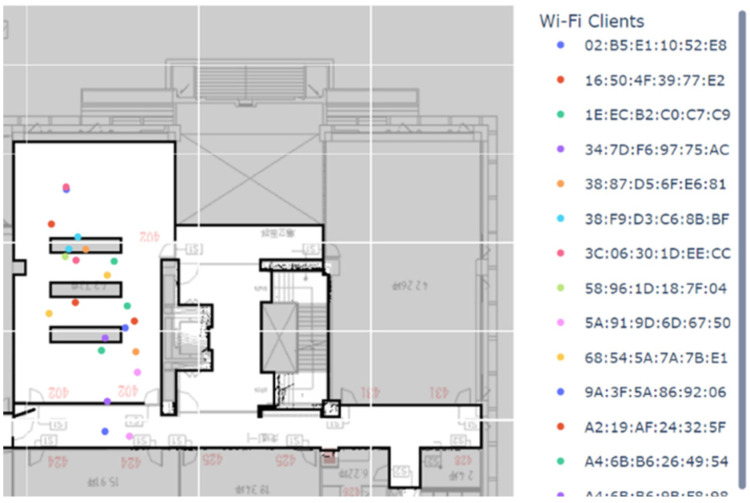
The screenshot of the Web plot of the online system.

The position estimate for each DUT is calculated on the server using WKNN, and a real-time Web plot is generated using Plotly Express and Dash. Figure 17 shows that the system can detect more than 20 connected Wi-Fi DUTs in a classroom. Each coloured dot represents the position estimate for an online DUT, and the MAC address of each is shown on the right side of the figure.

WKNN is a simple algorithm and a lazy learner, so the algorithm does not use the training data for any generalisation. It compares each individual data point when new online query data are supplied so the time it takes to predict the positions will increase with the number of online DUTs [35]. The system requires around 2 seconds to calculate the position estimates for 23 DUTs.

Previous studies [23,36] show that different models of DUTs perform differently in terms of positioning because each DUT has a different RSSI distribution. This occurs because different DUTs have different characteristics, such as the performance of the Wi-Fi antenna. The DUT does not actively participate in the positioning process so fewer Wi-Fi packets are transmitted if a phone screen is off or the device is in power-saving mode. Sniffers can then miss packets, so the DUT appears to be static in relation to one RP in a period of time. Complementary features, such as pedestrian dead reckoning (PDR), cannot be used for passive positioning, so mobility prediction using the speed and direction of travel for the DUT cannot be implemented [37,38].

## 8. Conclusions

A passive IPS is presented in this study, that uses Wi-Fi signals to determine the location of a DUT without requiring additional APIs or active participation from the user. The system employs Wi-Fi Sniffers to scan and collect the DUT’s RSSI as Wi-Fi fingerprints which are then used to map vectors of RSSI to RPs in the physical world. The online positioning is obtained using the WKNN method. Nonetheless, the impact of the optimal deployment of Sniffers on other positioning algorithms, such as random forest and deep learning, deserves to be explored in future work.

In order to maximise the Wi-Fi RSSI received at each RP for system performance improvement, a modified GA with an entropy-enhanced objective function is utilised to optimise the deployment of the Wi-Fi Sniffers. Data collection of RSSI at each RP is automated through a surveying robot for the Wi-Fi 2.4 GHz and 5 GHz bands. A configuration of 20 Wi-Fi Sniffers that are calculated using entropy enhancement for both bands allows passive positioning with a respective RMSE value of 2.41 m and 2.23 m. These values are at least 7.10% less than the values that are calculated without using entropy. Simulation results also demonstrated that the RMSE for positioning for the configuration that is calculated using entropy for both Wi-Fi bands is at least 9.02% less than that of the figure for a configuration that is calculated without using entropy for different numbers of Wi-Fi Sniffers. The increase in performance is verified by the measurement data for configurations that use 10, 15 and 20 Sniffers.

The study demonstrates a proof-of-concept real-time online passive IPS at NTU, which can detect the online presence of DUTs and use their RSSIs as online fingerprints for position estimation. In the experiment, wireless Sniffers are deployed to record the received signal strength indicator (RSSI) values of the device-under-test (DUT). For passive positioning systems, the AP-Sniffer network is specifically designed for positioning so the physical locations must be robust to physical tampering to ensure optimal Wi-Fi-based positioning. The method of previous studies [39,40,41], which monitors abnormal CSI waveforms, can be used by this study to detect any physical tampering with Sniffers to maintain good performance.

The system must restrict specific experimental areas and construct blacklists and whitelists to eliminate unwanted static targets, such as Wi-Fi printers or other Wi-Fi APs. This reduces the processing load for the server. Overall, this Internet of Things (IoT) solution effectively deploys wireless Sniffers using entropy-enhanced genetic algorithms to implement a passive fingerprinting positioning system, resulting in improved positioning accuracy.

## Figures and Tables

**Table 1:** Parameters used in genetic algorithm.

Parameters	Value
Number of possible candidate locations for Sniffers, R	133
Number of Sniffers required to be selected, M	20
Crossover rate	0.8
Mutation rate	0.02
Population size	200
Number of generations	600
RSSI max, $RSSI_{max}$	−30 dB
RSSI min, $RSSI_{min}$	−80 dB

**Table 2:** RMSE for simulation and measurement data (20 Wi-Fi Sniffers).

Dataset ( K = 10, 20 Wi-Fi Sniffers)	w/o Entropy (2.4 GHz)	w/ Entropy (2.4 GHz)	w/o Entropy (5 GHz)	w/ Entropy (5 GHz)
Simulation	2.109 m	1.705 m	1.778 m	1.518 m
Measurement	2.593 m	2.409 m	2.961 m	2.229 m

## Data Availability

Not applicable.
